# Luther Tucker and Son

**Published:** 1861-03

**Authors:** 


**LUTHER TUCKER AND SON**, Albany, N. Y., have issued No. 7 of their *Illustrated Annual Register and Almanac of Rural Affairs for* 1861, for 25 cents, full of practical information for farmers, gardeners, horticulturists, vine-dressers, fruiterers and poultry-raisers, and those laying out lawns, grounds and farms.

SINGLE numbers of HALL'S JOURNAL OF HEALTH may be had in Philadelphia of William P. Mowry, Esq., Post-Office entrance. Odd numbers for 1860, which subscribers did not receive through the post-office, will be supplied without charge. ☞ Our book on "SLEEP," with the JOURNAL OF HEALTH and the FIRESIDE MONTHLY for 1861, will be furnished for three dollars, or two dollars in addition to any present subscriber to the JOURNAL OF HEALTH. The reception of this JOURNAL or the FIRESIDE MONTHLY, after being ordered with the money, is evidence that the subscription was received.

